# Epidemiological and clinical analysis, and outcomes of tuberculosis co-infection among people living with HIV in Türkiye (2014–2024) ClinSurv HIV cohort: A large case series

**DOI:** 10.1371/journal.pone.0329267

**Published:** 2025-08-01

**Authors:** Ali Mert, Okan Derin, Esra Zerdali, Abdurrahman Kaya, Özlem Gül, Deniz Borcak, Ahmet Furkan Kurt, Meliha Meriç Koç, Bircan Kayaaslan, Ayşe Batırel, Alper Gündüz, İnci Yılmaz Nakir, Gülşen Yörük, Dilek Yıldız Sevgi, Hayat Kumbasar Karaosmanoğlu, Bilgül Mete, Melike Nur Özçelik, Nagehan D. Sarı, Yasemin Akkoyunlu, Fehmi Tabak

**Affiliations:** 1 Internal Medicine Department, Faculty of Medicine, Istanbul Medipol University, İstanbul, Türkiye; 2 Infectious Diseases and Clinical Microbiology Department, Istanbul Şişli Hamidiye Etfal Training and Research Hospital, İstanbul, Türkiye; 3 Epidemiology PhD Program, Graduate School of Health Sciences, Istanbul Medipol University, İstanbul, Türkiye; 4 Infectious Diseases and Clinical Microbiology Department, Istanbul Haseki Training and Research Hospital, İstanbul, Türkiye; 5 Infectious Diseases and Clinical Microbiology Department, Istanbul Training and Research Hospital, İstanbul, Türkiye; 6 Infectious Diseases and Clinical Microbiology Department, Istanbul Bakırköy Training and Research Hospital, İstanbul, Türkiye; 7 Infectious Diseases and Clinical Microbiology Department, Cerrahpaşa Faculty of Medicine, Istanbul University-Cerrahpaşa, İstanbul, Türkiye; 8 Infectious Diseases and Clinical Microbiology Department, Istanbul Bezmialem Vakif University, İstanbul, Türkiye; 9 Infectious Diseases and Clinical Microbiology Department, Hamidiye Faculty of Medicine, University of Health Sciences, İstanbul, Türkiye; 10 Infectious Diseases and Clinical Microbiology Department, Istanbul Çam ve Sakura City Hospital, İstanbul, Türkiye,; 11 Infectious Diseases and Clinical Microbiology Department, Ankara Bilkent City Hospital, Ankara, Türkiye; 12 Infectious Diseases and Clinical Microbiology Department, Faculty of Medicine, Ankara Yıldırım Beyazıt University, Ankara, Türkiye; 13 Infectious Diseases and Clinical Microbiology Department, Istanbul Kartal Dr. Lütfi Kırdar City Hospital, İstanbul, Türkiye; 14 Infectious Diseases and Clinical Microbiology Department, International Faculty of Medicine, University of Health Sciences, İstanbul, Türkiye; Management Sciences for Health (MSH), ETHIOPIA

## Abstract

**Background:**

Tuberculosis (TB) is one of the most common opportunistic infections in people living with HIV (PLHIV). Mycobacterium tuberculosis may cause more TB in all stages of HIV infection than in the general population, with the incidence of TB and the spread of pulmonary TB to other organs increasing as the CD4 count decreases.

**Objective:**

In this HIV cohort study, we aimed to evaluate the clinical features, diagnosis, and prognosis of TB among PLHIV in Türkiye.

**Materials and methods:**

We conducted a retrospective cohort study to analyze clinical outcomes and identify determinants of mortality among people living with HIV (PLHIV) co-infected with tuberculosis. We included 264 patients diagnosed and treated for TB across six centers in Türkiye. We extracted clinical, demographic, laboratory, microbiological, and radiological data from patient medical records. To identify independent predictors of mortality, we performed multivariable logistic regression and reported the results as odds ratios (ORs) with 95% confidence intervals (CIs).

**Results:**

Of the 9,687 PLHIV who were followed for 10 years, 2.7% (264 individuals) developed TB. The median age of these individuals was 40 years, and 89% were male.

The prevalence of pulmonary TB only, extrapulmonary TB only, and the coexistence of pulmonary and extrapulmonary TB were 42.4%, 48.8%, and 8.7%, respectively. Opportunistic infections and cancers were found in 23% (62 out of 264) of patients with HIV/TB co-infection. Among patients with HIV/TB co-infection, 42% showed lymphadenopathy, with 70% of these cases being generalized. In patients who underwent chest CT scans (n=200), radiological patterns revealed post primary TB in 46%, primary TB in 36%, and miliary TB in 18%. The positivity rates of Ehrlich-Ziehl-Neelsen staining (EZN), polymerase chain reaction (PCR), and TB cultures in clinical samples were found to be 47.5%, 72.5%, and 53%, respectively. Most of our patients (95%) were given the standard TB treatment regimen (HRZE), with a paradoxical reaction observed in 11.6% of cases and hepatotoxicity occurring in 18% of cases. Age, CD4 count (<200 cells/mm^3^-late presenters), and thrombocytopenia were identified as independent risk factors for mortality in the 58 patients (22%) who died after diagnosis.

**Conclusion:**

Even today, more than one fifth of patients with HIV–TB co-infection in our cohort died. Mortality was higher among individuals who presented late with tuberculosis disease, especially those with advanced immunosuppression (CD4 <200 cells/μL). These findings underscore the urgent need for early HIV diagnosis and systematic TB screening to reduce co-infection–related mortality and improve clinical outcomes.

## Introduction

Studies of human bones have shown that tuberculosis (TB) is an ancient disease dating back thousands of years, and its cause remained unknown until the discovery of *Mycobacterium tuberculosis* (*M. tuberculosis*) by Robert Koch in 1882 [1,2]. Although global incidence is declining, tuberculosis remains a major public health issue, particularly in sub-Saharan Africa. Each year, it affects around 10 million people and causes 1.5 million deaths, making it the leading cause of death from an infectious disease. Even among untreated HIV-negative individuals, pulmonary tuberculosis has a high mortality rate of approximately 50%. However, with the World Health Organization (WHO)’s recommended 6-month anti-TB regimen, about 85% of cases can be successfully cured [3]. Tuberculosis co-infection in people living with HIV (PLHIV) remains a major public health challenge. According to the WHO Global TB Report 2023, an estimated 10.6 million new TB cases occurred in 2022, 7% of which were HIV-positive. Of the 1.3 million TB-related deaths, 13% (167,000) occurred among PLHIV [4]. In 2014, approximately one-quarter of the global population—around 1.7 billion people—was estimated to have tuberculosis infection, previously termed latent tuberculosis infection (LTBI) [5]. The lifetime risk of TB infection progressing to TB disease (previously termed active TB) in immunocompetent individuals is approximately 10% [6]. In PLHIV with TB infection, TB disease develops at approximately 10% (3–21%) per year, with HIV being the most significant risk factor for progression to disease [7]. In patients with TB infection, TB reactivation risk increases with worsening immunodeficiency. Even with effective ART, PLHIV remains at higher TB risk than the general population. While TB was previously the leading infectious cause of death worldwide, HIV remains the most significant risk factor for active TB development.

In our country, while TB incidence has steadily declined (10 cases per 100,000 population in 2020), newly diagnosed HIV cases have surged from 2,687 in 2016–5,710 in 2022. As of 2021, HIV prevalence among TB patients was estimated at 1% [8]. Our review of the English-language medical literature (PubMed) found no large-scale studies on HIV/AIDS-TB co-infection conducted in our country.

This study was the first large-scale investigation of tuberculosis co-infection among PLHIV in Türkiye. We evaluated the clinical characteristics, diagnostic approaches, and outcomes of TB in PLHIV. We assessed co-infection risk, emphasized the importance of early diagnosis and treatment in reducing mortality and transmission, and examined the concurrent use of ART and anti-TB therapy. Given the persistence of high mortality despite the availability of effective treatments, we also explored potential underlying causes.

## Methodology

### Study design and participants

This study employed a retrospective cohort design to analyze clinical outcomes and survival determinants in people living with HIV (PLHIV) who developed tuberculosis. The analysis was based on a subset of the national ClinSurv HIV cohort, which includes 9,687 PLHIV enrolled across multiple centers in Türkiye. For this study, we included 264 individuals diagnosed and treated for TB between January 2014 and March 2024 at six participating centers. Inclusion criteria required patients to be aged 18 years or older, have confirmed TB, and possess complete medical records available for review.

### Case definition

Criteria for TB diagnosis in PLHIV included at least one of the following, alongside strong clinical evidence consistent with active TB — such as current cough [9] or coughing lasting more than two weeks [10,11], fever, night sweats, anorexia, weight loss, fatigue, headache, mental changes, chest pain, lymphadenopathy, or relevant radiological findings: 1) Microbiological criterion, 2) Pathological criterion (histologic evidence of caseating or noncaseating necrotizing granulomas), 3) Pathological criterion + microbiological criterion. Microbiological criterion was established by acid-fast bacilli (AFB) smear, nucleic acid amplification (NAA) testing (GeneXpert MTB/RIF and GeneXpert MTB/RIF Ultra), and isolation of *M. tuberculosis* from a bodily fluid (e.g., the culture of sputum, bronchoalveolar lavage, or pleural-pericardial-peritoneal fluid) or tissue (e.g., biopsy) [9–11]. The Ehrlich-Ziehl-Neelsen (EZN) method was used to AFB from clinical samples (fluid or solid tissue). *M. tuberculosis* was cultured in Löwenstein–Jensen (LJ) media and Mycobacterial Growth Indicator Tube (MGIT, Becton Dickinson). *M. tuberculosis* DNA was investigated by polymerase chain reaction (PCR). The clinical forms of TB were classified according to CDC criteria as follows: pulmonary TB only, extrapulmonary TB (EPTB) only, and combined pulmonary and extrapulmonary TB [12].

Anti-TB drug combinations, their side effects, and complications of the disease were also noted. The diagnosis of hepatotoxicity from anti-TB drugs was established by the presence of at least one of the following: In cases where aminotransferase is five or more times higher than the upper limit of normal (with or without symptoms), or three or more times higher in the presence of symptoms or jaundice (i.e., bilirubin >3 mg/dL) [13].

Patients fulfilling the diagnostic criteria of fever of unknown origin (FUO) and paradoxical reaction were also determined [14,15]. FUO is characterized by a fever higher than 38.3°C on several occasions, persisting without a diagnosis for at least 3 weeks despite at least 1 week of investigation in the hospital. Paradoxical reaction is defined as immune reconstitution inflammatory syndrome (IRIS) after the initiation of antiretroviral therapy (ART) in patients being treated for TB. Paradoxical reaction refers to the transient worsening of a pre-existing tuberculous lesion or the development of new lesions during proper anti-TB therapy.

### Data collection

Clinical, demographic, laboratory, microbiological, and radiological data were extracted, including age, sex, ethnicity, TB type (pulmonary or extrapulmonary), HIV status (CD4 count, viral load), and treatment outcomes. Treatment details, including anti-TB and ART types, were collected. The primary outcome was survival status at the end of follow-up. Tuberculin skin tests (TST) and interferon-γ release assays (IGRAs) were recorded. TST induration was measured in millimeters after 48–72 hours by experienced staff, with ≥5 mm considered positive in immunosuppressed patients. Serology results (HBsAg, anti-HCV, syphilis) were also included.

### Statistical analysis

Data were analyzed using R statistical software. Descriptive statistics (mean, standard deviation, frequencies, and percentages) were used to summarize the characteristics of the participants. Differences in survival outcomes were evaluated using Chi-squared tests for independence between categorical variables and Wilcoxon rank-sum tests for comparing distributions of continuous variables. Multivariable logistic regression was performed to identify independent predictors of mortality, with results expressed in adjusted odds ratios (aORs) and 95% confidence intervals (CIs). The final model was developed using a stepwise logistic regression approach, considering both forward and backward selection. The fit of the final model was evaluated using the residual deviance and the Akaike Information Criterion (AIC). Missing data were handled using complete case analysis, where only records with no missing values for relevant variables were included in the analysis.

## Results

### Participant characteristics

Over the past decade, TB occurred in 264 (2.7%) of 9,687 PLHIV under follow-up. Among the 264 patients, 58 (22%) died, while 206 (78%) survived. The median age at TB diagnosis was 40 years (IQR: 32–48), with deceased patients being older than survivors (median ages: 44, IQR: 32–52 vs. 39, IQR: 32–46; p = 0.017). Most patients were male (89%) and of Türkiye origin (91%). Clinical and laboratory characteristics at admission are summarized in Tables 1 and 2.

**Table 1:** Baseline characteristics of patients with HIV–TB Co-infection, stratified by mortality outcome.

Characteristic	Overall, N = 264^1^	Deceased, N = 58^1^	Survived, N = 206^1^	p-value^2^
**Age**	40 (32, 48)	44 (32, 52)	39 (32, 46)	0.017
**Male Sex**	235 (89%)	51 (88%)	184 (89%)	0.8
**Nationality**				0.9
Turkish	239 (91%)	53 (91%)	186 (91%)	
Non-Turkish	24 (9.0%)	5 (8.6%)	19 (9.3%)	
**Transmission route**				0.017
Heterosexual	130 (63%)	31 (72%)	99 (60%)	
Homosexual	54 (26%)	6 (14%)	48 (29%)	
Bisexual	22 (11%)	4 (9.3%)	18 (11%)	
Parenteral	2 (1.0%)	2 (4.7%)	0 (0%)	
**Clinical manifestations**				
Cough	161 (61%)	38 (66%)	123 (60%)	0.4
Fever	191 (72%)	51 (88%)	140 (68%)	0.003
FUO	90 (34%)	23 (40%)	67 (33%)	0.3
Fatigue	241 (91%)	53 (91%)	188 (91%)	>0.9
Anorexia	225 (85%)	54 (93%)	171 (83%)	0.056
Weight loss	206 (78%)	52 (90%)	154 (75%)	0.015
Headache	54 (20%)	20 (34%)	34 (17%)	0.003
Mental changes	41 (16%)	16 (28%)	25 (12%)	0.004
Hemoptysis	13 (4.9%)	3 (5.2%)	10 (4.9%)	>0.9
Dyspnea	53 (20%)	19 (33%)	34 (17%)	0.006
Chest pain	38 (14%)	12 (21%)	26 (13%)	0.12
Neck stiffness	19 (7.2%)	8 (14%)	11 (5.3%)	0.041
**Lymphadenopathy**				0.029
Generalized	69 (67%)	19 (86%)	50 (62%)	
Localized	34 (33%)	3 (14%)	31 (38%)	
**Clinical form**				0.2
Only extrapulmonary	129 (49%)	34 (59%)	95 (46%)	
Only pulmonary	112 (42%)	20 (34%)	92 (45%)	
Pulmonary and Extrapulmonary	23 (8.7%)	4 (6.9%)	19 (9.2%)	

^1^n (%); Median (IQR); Range, ^2^Wilcoxon rank sum test; Pearson’s Chi-squared test; Fisher’s exact test.

**Table 2:** Summary of baseline laboratory findings of HIV/TB co-infected patients.

Characteristic	Overall, N = 264^1^	Deceased, N = 58^1^	Survived, N = 206^1^	p-value^2^
**Hematological**				
Anemia (Hgb male < 13g/dL, female <12)	205 (79%)	54 (95%)	151 (74%)	<0.001
Leukopenia <4.000/ml	73 (28%)	21 (37%)	52 (25%)	0.091
Lymphopenia <1000/ml	129 (50%)	38 (68%)	91 (45%)	0.002
CD4 count <200 cells/mm^3^,	182 (71%)	46 (85%)	136 (67%)	0.010
Thrombocytopenia <150.000/ml Pancytopenia	63 (24%) 33(12.5%)	22 (39%)	41 (20%)	0.004
**Biochemistry**				
Elevated transaminases	48 (19%)	13 (23%)	35 (17%)	0.4
Hypoalbuminemia (<3.5 g/dl)	139 (53%)	43 (75%)	96 (47%)	<0.001
Hyperbilirubinemia	24 (9.4%)	6 (11%)	18 (9.0%)	0.7
**CRP**				0.004
Normal	34 (13%)	4 (7.1%)	30 (15%)	
<10 times increase	115 (45%)	20 (36%)	95 (48%)	
10-20 times increase	43 (17%)	8 (14%)	35 (18%)	
>20 times increase	63 (25%)	24 (43%)	39 (20%)	
**ESR**				0.13
<20mm/h	19 (7.7%)	2 (3.7%)	17 (8.8%)	
20-49 mm/h	54 (22%)	7 (13%)	47 (24%)	
50-99 mm/h	127 (51%)	34 (63%)	93 (48%)	
>100 mm/h	47 (19%)	11 (20%)	36 (19%)	
**TST positive**	38			>0.9
CD4 count <200 cells/mm^3^	20 (53%)	2 (50%)	18 (53%)	
CD4 count ≥200 cells/mm^3^	18 (47%)	2 (50%)	16 (47%)	
**IGRA positive**				
CD4 count <200 cells/mm^3^	8 (80%)	4 (100%)	4 (67%)	0.5
CD4 count ≥200 cells/mm^3^	2 (20%)	0 (0%)	2 (33%)	

^1^n (%); Median (IQR); Range; ^2^Wilcoxon rank sum test; Pearson’s Chi-squared test; Fisher’s exact test.

TST: Tuberculin Skin Test, CRP: C- Reactive Protein, ESR: Erythrocyte Sedimentation Rate, IGRA: Interferon Gamma Release Assay.

**Fig 1:**
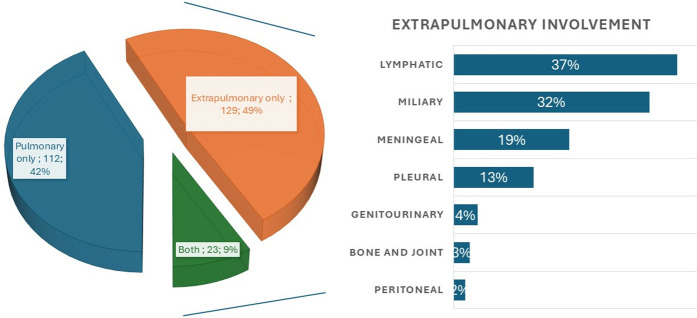
TB clinical form distribution in patients with HIV/TB co-infection (n = 264).

Lymphadenopathy (LAP) was present in 42% (110), with 70% showing generalized LAP and 30% localized LAP (Table 3). TB clinical forms are shown in Fig 1. Opportunistic infections and malignancies were observed in 23% (n = 62) of HIV/TB co-infected patients and are detailed in Table 4.

**Table 3:** Localization of lymphadenopathy in patients with HIV/TB co-infection (n = 110/264) (42%).

I-Generalized LAP	75 (70%)
II-Localized LAP	35 (30%)
Peripheral LAP	
1-Cervical	63
2-Supraclavicular	23
3-Axiliary	31
4-Inguinal	18
Mediastinal LAP	79
1-Bilateral hilar	24
2-Left hilar	2
3-Right hilar	1
Abdominal LAP	45

LAP: Lymphadenopathy.

**Table 4:** Opportunistic infections and cancers in patients with HIV/TB co-infection (n = 62/264) (%23).

Factor	N(%)
Pneumocystis jirovecii pneumonia (PJP)	19 (7)
CMV retinitis	6 (2)
Toxoplasma gondii encephalitis	6 (2)
Cryptococcal meningitis	5 (2)
Kaposi’s sarcoma	13 (5)
Lymphoma	13 (5)
Total	62(23)

Positivity rates for EZN, PCR, and TB cultures in clinical samples were 47.5%, 88%, and 56%, respectively (Table 5). Tissue biopsy granulomas (n = 70) showed necrosis in 71% (50/70) and caseous necrosis in 17% (12/70). HBsAg, anti-HCV, and syphilis serology positivity rates were 9% (24/261), 3% (8/259), and 15% (n = 38/253), respectively.

**Table 5:** Microbiological studies of clinical samples from HIV-TB co-infected patients.

Sample	EZN positivity ratio n (%)	PCR positivity rate n (%)	TB culture positivity rate n (%)
Sputum	70 (NA)	53 (NA)	98 (64)
Sterile body fluid	10 (NA)	11 (NA)	11 (NA)
Cerebrospinal fluid	4 (NA)	12 (NA)	4(NA)
Tissue biopsy	31 (NA)	13 (NA)	22 (NA)
Total	115/242 (47.5)	89/101 (88)	135/239 (56)

NA: During the data collection process, the sample types for negative results were not specified for certain samples. Consequently, test positivity ratios could not be calculated for individual sample types, except for sputum culture. However, as the positivity ratio for each test was collected, an overall positivity ratio can still be presented.

### Clinical and treatment outcomes

Of the TB diagnoses, 51% were pulmonary, 49% extrapulmonary, and 8.7% involved both forms. Miliary TB was more common in deceased patients (29% vs. 16% in survivors; p = 0.017). Deceased patients had a significantly lower median CD4 count at TB diagnosis (55 vs. 100 cells/μL; p = 0.001) and higher HIV viral loads (median: 635,697 vs. 373,366 copies/mL; p = 0.13).

Survivors had a longer median anti-TB treatment duration than those who died (9 vs. 4 months; p < 0.001). Among 242 patients with available ART regimen data, the most common regimen was dolutegravir plus tenofovir disoproxil fumarate/emtricitabine (DTG + TDF/FTC), used in 53.7% of cases. Efavirenz-based regimens (EFV + TDF/FTC) and lopinavir/ritonavir + TDF/FTC were used in 23.6% and 10.3% of patients, respectively. Among 202 patients with known ART timing, the median interval from anti-TB treatment initiation to ART initiation was 3 weeks (IQR: 2–5), and 13 patients (6.4%) were already on ART at the time of TB diagnosis. ART timing did not differ significantly between deceased and surviving patients (2.5 vs. 3 weeks; p = 0.5). Among patients with TB meningitis (n = 29), ART was initiated at a median of 4.5 weeks (IQR: 3–9), compared to 3.0 weeks (IQR: 2–5.5) in those without meningitis, with no statistically significant difference (p = 0.239).

### Predictors of mortality

The univariable and multivariable analyses of factors associated with mortality in HIV/TB co-infected patients are summarized in Table 6. Univariable analysis identified several significant mortality risk factors, including older age (OR: 1.04, 95% CI: 1.01, 1.07), fever (OR: 3.43, 95% CI: 1.57,8.66), dyspnea (OR: 2.46, 95% CI: 1.26, 4.75), altered consciousness (OR: 2.76, 95% CI: 1.34, 5.59), weight loss (OR: 2.93, 95% CI: 1.27, 7.96), disseminated LAP (OR: 3.93, 95% CI: 1.21,17.7), and CD4 count <200 cells/mm^3^ (OR: 2.79, 95% CI: 1.31,6.69). Thrombocytopenia (OR: 2.48, 95% CI: 1.31, 4.67) and pancytopenia (OR: 3.29, 95% CI: 1.54, 6.94) were also associated with increased mortality.

**Table 6:** Univariable and multivariable analysis of factors on mortality.

Characteristic	Univariable Analysis				Multivariable Analysis		
	N	OR^*1*^	95% CI^1^	p-value	aOR^1^	95% CI^1^	p-value
**Age**	264	1.04	1.01, 1.07	0.003	1.05	1.02, 1.09	0.002
**Female sex**	264	1.15	0.43, 2.72	0.8			
**Fever**	264	3.43	1.57,8.66	0.004	2.56	0.94,7.95	0.08
**Dyspnea**	264	2.46	1.26, 4.75	0.007	1.51	0.64,3.45	0.3
**Hemoptysis**	264	1.07	0.23, 3.64	>0.9			
**Altered consciousness**	264	2.76	1.34, 5.59	0.005	1.80	0.65,5.02	0.3
**TB Meningitis**	264	2.05	0.87, 4.62	0.089			
**Weight loss**	264	2.93	1.27, 7.96	0.020	2.73	0.84,10.9	0.12
**Disseminated Lymphadenopathy**	103	3.93	1.21,17.7	0.039			
**Time between TB and HIV diagnosis**	264	1.00	0.99, 1.01	>0.9			
**Clinical Form**	264						
**Pulmonary**		—	—		—	—	
**Extrapulmonary**		1.65	0.89,3.11	0.12	1.26	0.56,2.89	0.6
**Pulmonary and Extrapulmonary**		0.97	0.26,2.92	>0.9	0.51	0.10,2.08	0.4
**Miliary Involvement**	264	2.25	1.13, 4.42	0.019	1.01	0.40,2.42	>0.9
**Paradox reaction**	249	2.02	0.82, 4.68	0.11			
**Concomitant Malignity (Kaposi and other hematological malignities)**	264	2.18	0.95, 4.79	0.057			
**Opportunistic infection**	264	1.18	0.54, 2.45	0.7			
**Leukopenia**	261	1.71	0.90,3.16	0.09			
**Lymphopenia**	259	2.60	1.41,4.95	0.003	1.19	0.51,2.80	0.3
**Thrombocytopenia**	260	2.48	1.31, 4.67	0.005	2.54	1.10,5.90	0.03
**Pancytopenia**	261	3.29	1.54, 6.94	0.002			
**CD4** ^** + **^ **T cell <200/mm** ^**3**^	256	2.79	1.31,6.69	0.013	15.6*	1.85,186	0.02
**Transmission route: Other than homosexual**	264	2.26	1.12,4.99	0.03			
**Hyperbilirubinemia**	256	1.18	0.41,2.99	0.7			
**Hypoalbuminemia**	261	3.46	1.82,6.91	<0.001	2.09	0.88,5.19	0.10
**C Reactive Protein (CRP)**	255	1.00	1.00, 1.00	>0.9			
**Erythrocyte Sedimentation Rate (ESR)**	247	1.01	1.00, 1.02	0.013	1.01	0.99,1.02	0.4
**Hepatotoxicity**	264	1.67	0.80, 3.34	0.2			

^1^OR = Odds Ratio, CI = Confidence Interval; * There is an interaction between CD4^+^ T cell count and transmission route (OR was achieved after interaction term).

Multivariable analysis confirmed older age (OR: 1.05, 95% CI: 1.02, 1.09), thrombocytopenia (OR: 2.54, 95% CI: 1.10,5.90), and low CD4 count, especially with non-homosexual transmission routes (OR: 15.6, 95% CI: 1.85,186), as independent mortality predictors.

The interaction between CD4 count and transmission route highlighted the increased effect of immune suppression in non-homosexual transmission. In our analysis of mortality factors in HIV/TB co-infected patients, significant differences in clinical and demographic variables were noted across transmission routes (Table 6). A CD4 count <200 cells/mm^3^ had an OR of 2.79 for mortality in univariable analysis, which shifted to 0.18 in multivariable analysis, prompting further investigation of interactions with other variables, particularly HIV transmission routes. The interaction analysis revealed that a CD4 count <200 cells/mm^3^ in non-homosexual transmission routes was associated with a markedly higher OR of 15.6 for mortality. The non-homosexual group had older median age and more severe inflammatory markers at diagnosis. Median age of deceased patients was 46 years (IQR: 33–54) in the non-homosexual group versus 37 years (IQR: 31–50) in the homosexual group, suggesting age as a contributing factor to higher mortality. Details of the multivariable model and interaction terms are provided in supporting file (S1 File).

Inflammatory markers were also elevated in the non-homosexual group, with median CRP levels of 85 mg/L compared to 29 mg/L in the homosexual group and median ESR of 90 mm/h versus 67 mm/h.

## Discussion

In this study, TB developed in 3% of PLHIV, with 90% of cases being male. Half had extrapulmonary TB, and lymphadenopathy was present in 50%, two-thirds of whom had systemic lymphadenitis. Post-primary pulmonary TB was the most common presentation, identified in about half of the patients, followed by primary TB in nearly a third, and miliary TB in approximately one-fifth. EZN and TB culture positivity were observed in half of the samples, while PCR was positive in 75%. Nearly all patients received the standard TB regimen, with paradoxical reactions in 10% and hepatotoxicity in 20%. Age, CD4 lymphopenia, and thrombocytopenia were risk factors for mortality, which occurred in 20% of cases.

Opportunistic infections (OIs) are common and a leading cause of death in advanced HIV/AIDS patients. TB, prevalent in all stages of HIV infection, is a frequent OI. A southern China study of hospitalized HIV patients (60% with AIDS) found 71% (n = 8,982/12,612) developed OIs, with TB in 35% [16]. A Turkish systematic review showed a 17% OI rate in PLHIV, with TB at 5.5% [17]. This HIV cohort study found TB in 2.7% of PLHIV, with OIs in roughly 25% of HIV/AIDS-TB co-infections. These findings highlight the persistent burden of TB as an OI in PLHIV, emphasizing the need for early detection and comprehensive management strategies.

Before 2010, TB diagnosis primarily relied on EZN staining and cultures, which had limitations: low sensitivity for AFB smears and slow results for cultures. FDA-approved NAA tests (Xpert MTB/RIF in 2010 and Xpert MTB/RIF Ultra in 2017) reduced diagnosis time and rifampicin resistance determination to 2 hours, leading to WHO recommendations for their use [18,19]. Sputum AFB microscopy has low sensitivity (62%) but high specificity (100%) [20]. Xpert MTB/RIF and Ultra show higher sputum sensitivity (75% and 88%) and specificity (100% and 93%) [21]. However, sensitivity varies for extrapulmonary TB (EPTB) due to lower bacillus loads in fluid/tissue samples. A meta-analysis found EPTB sensitivity for Xpert MTB/RIF or Ultra at 50–90% and specificity >90% [22]. In this study, PCR testing of 101 diverse clinical samples, including sputum, fluid, and tissue specimens, demonstrated an 88% positivity rate for the M. tuberculosis complex. These findings highlight the ongoing need for advanced molecular diagnostics, particularly for EPTB, where traditional methods remain less effective.

TSTs and IGRAs, reflecting adaptive immune responses to mycobacterial antigens, indicate TB exposure but don’t diagnose active disease. Test negativity (anergy) is more likely with lower CD4 counts [23]. Of 87 patients tested (66 TST, 21 IGRA), the combined positivity rate was 55% (n = 48). Positivity was 44% (n = 28) in those with CD4 counts <200 and 83% (n = 20) in those with CD4 counts ≥200.

In the USA in 2022, most TB cases were PTB only (70.4%), followed by EPTB only (18.8%), and both (10.6%) [12]. Of the 8925 TB cases reported in Türkiye in 2020, 65% (n = 5802) were PTB, and 35% (n = 3123) were EPTB [8]. Lower CD4 counts (<200) in AIDS patients increase the likelihood of both PTB and EPTB [23]. Studies on HIV/AIDS-TB co-infection show this co-occurrence ranging from 28% to 90% [24–27]. In this study, we found 42.4% PTB only, 48.8% EPTB only, and 8.7% both. This lower co-occurrence rate compared to other studies may be due to the retrospective and heterogeneous nature of these studies, leading to varied distribution rates.

HIV infection alters PTB radiographic patterns based on immunodeficiency severity. Patients with CD4 ≥ 200 cells/mm^3^ typically present with upper lobe consolidation and cavitation, resembling TB in HIV-negative individuals, where post-primary PTB primarily affects the apical-posterior segment of the upper lobes, followed by the superior segment of the lower lobes and the anterior segment of the upper lobes [22,28]. Common findings include consolidation, often with cavitation, but without hilar or mediastinal adenopathy. Conversely, in AIDS patients, post-primary PTB often mimics primary TB radiographically, showing atypical patterns. These include middle or lower lung involvement, non-cavitating nodular opacities or consolidation, pleural effusions, and often hilar/mediastinal adenopathy. Upper lobe consolidation or cavitation is less common. In some cases, chest X-rays may even appear normal. Frey et al. found that adults with low CD4 counts (<200 cells/µL) are more frequently presented with mediastinal lymphadenopathy and miliary patterns, while cavitation and consolidation were less likely [29]. Greenberg et al. observed a range of radiographic presentations in AIDS patients with PTB, including primary TB patterns (36%), post-primary patterns (28%), atypical infiltrates (13%), minimal changes (5%), miliary patterns (3%), and normal radiographs (15%) [30]. Notably, about 15% of AIDS patients with confirmed PTB have negative chest X-rays, a rarity in non-AIDS patients [31,32]. Chest CT scans of 200 patients in this study revealed diverse radiological patterns. This study aligns with previous research, showing post-primary PTB in 46%, primary TB in 36%, and miliary TB in 18%. Lower CD4 counts (≤200) were associated with a higher prevalence of primary (39% vs 27%) and miliary TB (19% vs 16%). Given these variations, non-contrast chest CT is a valuable tool in detecting TB in PLHIV.

TB lymphadenitis (LAP) is the most frequent form of EPTB [23]. While typically affecting the cervical lymph nodes (often unilaterally), it can also occur in axillary, inguinal, and supraclavicular regions [32–34]. Clinical manifestations vary depending on the location and the patient’s immune status. In AIDS patients, TB LAP often involves a significant mycobacterial load, leading to systemic symptoms like fever, sweating, and weight loss. Generalized TB LAP (70%) is more common in AIDS patients, compared to 17%−35% in immunocompetent individuals, as seen in this study.

The standard treatment consists of isoniazid (H), rifampin (R), pyrazinamide (Z), and ethambutol (E). The standard treatment for pulmonary or extrapulmonary TB with susceptible pathogens is (2HRZE/4HR), lasting ≥6 months, regardless of age, disease severity, or HIV status [13]. The treatment regimen or duration, including induction and continuation phases, may be adjusted based on the site of involvement and drug resistance. However, recovery in HIV/AIDS-TB co-infection is challenging due to immunosuppression and treatment non-compliance [35,36]. Therefore, HIV screening is recommended for all TB patients, as HIV and TB co-infection are common, and HIV co-infection influences TB’s course and treatment. In 2022, the WHO recommended a 6-month bedaquiline, pretomanid, linezolid, and moxifloxacin regimen for most rifampin-resistant TB cases, including those with HIV [37]. While steroids are recommended for HIV-associated TB meningitis, they may not improve mortality or neurological outcomes [38]. Early ART initiation is crucial in HIV-TB co-infection to reduce HIV-related morbidity and mortality. The WHO recommends starting ART within 2 weeks of TB treatment, irrespective of CD4 count, except for TB meningitis cases, where a 4–8-week delay is advised [9]. Early ART initiation lowers mortality but may increase IRIS risk, especially with CD4 counts below 50 [39]. In this study, 95% (252/264) of patients received the standard HRZE regimen. Rifabutin was used instead of rifampin in five cases, while four cases required the addition of moxifloxacin. One patient with choroidal tubercles and visual impairment replaced ethambutol with moxifloxacin. Eight cases received MDR-TB treatment, and one patient died before treatment initiation.

Hepatotoxicity, a potential side effect of isoniazid, rifampin, or pyrazinamide, occurs in 8% to 28% of HIV/AIDS-TB patients undergoing treatment [40]. In this study, 18% (47/264) of patients developed hepatotoxicity, consistent with published data. Close monitoring of liver function tests and timely regimen adjustments are essential to minimize treatment interruptions and improve adherence.

In line with the EACS 2024 guidelines [41], ART should be initiated in all individuals with TB/HIV co-infection regardless of CD4 count, with earlier initiation (within 2 weeks) recommended for those with CD4 < 50 cells/µL, except in cases of TB meningitis where ART should be delayed by 4 weeks. In this cohort, the median ART initiation time was 3 weeks, and approximately 6% of patients were already on ART at the time of TB diagnosis. No significant difference was observed in ART timing between patients who died and those who survived, suggesting that ART initiation may have been appropriately individualized based on clinical presentation. Among patients with TB meningitis, the median ART initiation was slightly delayed (4.5 weeks vs. 3.0 weeks), though this difference was not statistically significant, possibly reflecting clinical caution due to IRIS risk. The most frequently used ART regimen was dolutegravir combined with TDF/FTC, consistent with guideline-based recommendations.

A meta-analysis found an 18% incidence of paradoxical TB-associated IRIS in HIV-TB patients starting ART [41]. Higher IRIS rates are linked to lower CD4 counts, high HIV viral loads at ART initiation, and shorter intervals between TB treatment and ART initiation. IRIS typically occurs within 60 days of starting ART, though the range varies from 10 to 180 days [15]. In this study, 11.6% (n = 29/250) of patients developed TB-IRIS, typically at a median of 4 weeks (IQR: 3.3 to 8.0) after starting TB treatment. Importantly, IRIS development did not increase mortality in the patients in this study.

An estimated 39 million people were living with HIV at the end of 2022, with 1.3 million new infections and 630,000 AIDS-related deaths globally [42]. TB is a leading cause of death in people co-infected with HIV and TB. Autopsy studies in high TB-burden African countries show TB, particularly disseminated TB, as the most common cause of death (69%−79%) in AIDS patients [43–46]. Mortality rates in HIV-TB co-infected patients during treatment range from 5% to 37.6% [47–54]. In a South African study, the incidence of TB was still four times higher among individuals with normalized CD4 counts on ART compared to those living in the same community who did not have HIV infection [55]. TB meningitis in HIV-infected individuals carries a high mortality rate, around 40% [56]. The mortality rate in this study was 22%, which is consistent with rates reported in similar studies [48–55]. In this study, surviving patients received a median of 9 months of anti-TB treatment, whereas those who died had received a median of 4 months at the time of death.

Several studies have identified key risk factors associated with increased mortality among patients with HIV/TB co-infection, including advanced age, tuberculous meningitis, hypoalbuminemia, anemia, low CD4 cell count, disseminated TB, absence of antiretroviral therapy (ART), and coexisting non-AIDS-related comorbidities [47–53]. The multivariable logistic regression analysis identified older age, low CD4 count (<200 cells/mm^3^), and thrombocytopenia as independent predictors of mortality. Notably, a significant interaction was observed between HIV transmission route and CD4 count, underscoring the role of late HIV diagnosis in adverse outcomes. This finding is consistent with previous studies suggesting that individuals infected through heterosexual transmission are more likely to present with advanced immunosuppression due to delayed diagnosis [57]. This is further supported by the elevated inflammatory markers (ESR and CRP) observed in the non-homosexual group, indicating more severe disease at presentation. These findings are consistent with the interaction effect identified in our analysis, wherein non-homosexual transmission routes were associated with a significantly increased risk of mortality, particularly in the context of advanced immunosuppression.

The combination of older age, elevated inflammatory markers, and severe immunosuppression among non-homosexual individuals underscores the need for targeted interventions. Efforts to reduce delays in HIV diagnosis and improve early engagement in care—particularly in heterosexual populations—may significantly improve clinical outcomes and reduce mortality in this vulnerable group.

This study highlights the critical importance of early diagnosis and timely treatment in improving outcomes and preventing TB transmission among PLHIV. Despite the availability of effective ART and anti-TB therapies, persistently high mortality rates point to delays in diagnosis. To mitigate this, the use of non-contrast thoracic CT imaging at initial assessment—particularly in asymptomatic individuals with normal lung exams but elevated inflammatory markers (CRP and/or ESR)—may aid in earlier TB detection. Furthermore, prophylactic treatment for individuals with latent TB infection remains essential in reducing progression to active disease.

This study has several strengths. First, the analysis was based on a subset of the national ClinSurv HIV cohort, which includes 9,687 people living with HIV (PLHIV) enrolled across multiple centers in Türkiye. From this cohort, we included 264 individuals diagnosed and treated for tuberculosis between January 2014 and March 2024—constituting one of the largest case series on TB co-infection among PLHIV in the country. Second, the multi-center design, with data collected from six geographically diverse centers, enhances the generalizability of the findings and reduces the potential for single-center bias. Third, the study incorporated a wide range of clinical, demographic, laboratory, microbiological, and radiological variables, allowing for a comprehensive and in-depth analysis of TB co-infection in PLHIV.

This study has several limitations. First, its retrospective design limits the ability to establish causal relationships between variables. Second, the analysis relies on the availability and completeness of medical records, which may introduce information bias. Third, as the study was conducted solely in Türkiye, the findings may not be fully generalizable to other settings with different epidemiological patterns or healthcare systems. Lastly, data on isoniazid preventive therapy (IPT) use were unavailable, as this information was not systematically collected across study sites, limiting the ability to evaluate the protective role of IPT against TB progression in this population.

In conclusion, even today, 22% of our patients with HIV-TB co-infection have died. Mortality is higher among those with AIDS-TB co-infection. Diagnosing HIV-infected patients before they progress to the AIDS stage and initiating ART appears to be among the most important strategies for preventing deaths from TB.

## Supporting information

S1 File Supporting material for logistic regression model development. (PDF)
