# Supplementary material for: Epidemiological and clinical analysis, and outcomes of tuberculosis co-infection among people living with HIV in Türkiye (2014–2024) ClinSurv HIV cohort: A large case series
Source: PLoS One. 2025 Aug 1;20(8):e0329267. doi: 10.1371/journal.pone.0329267 (PMC12316272; doi:10.1371/journal.pone.0329267)
Supplement: S1 File — (PDF) [file pone.0329267.s001.pdf]

S1. Supporting Material for Logistic Regression Model Development

Null Model (model0)

The null model includes only the intercept term, representing the baseline log-odds of the outcome (e.g., survival or death). In this case, the AUC is 0.5, indicating no predictive power, as expected since no predictors are included in the model (S1 Figure).

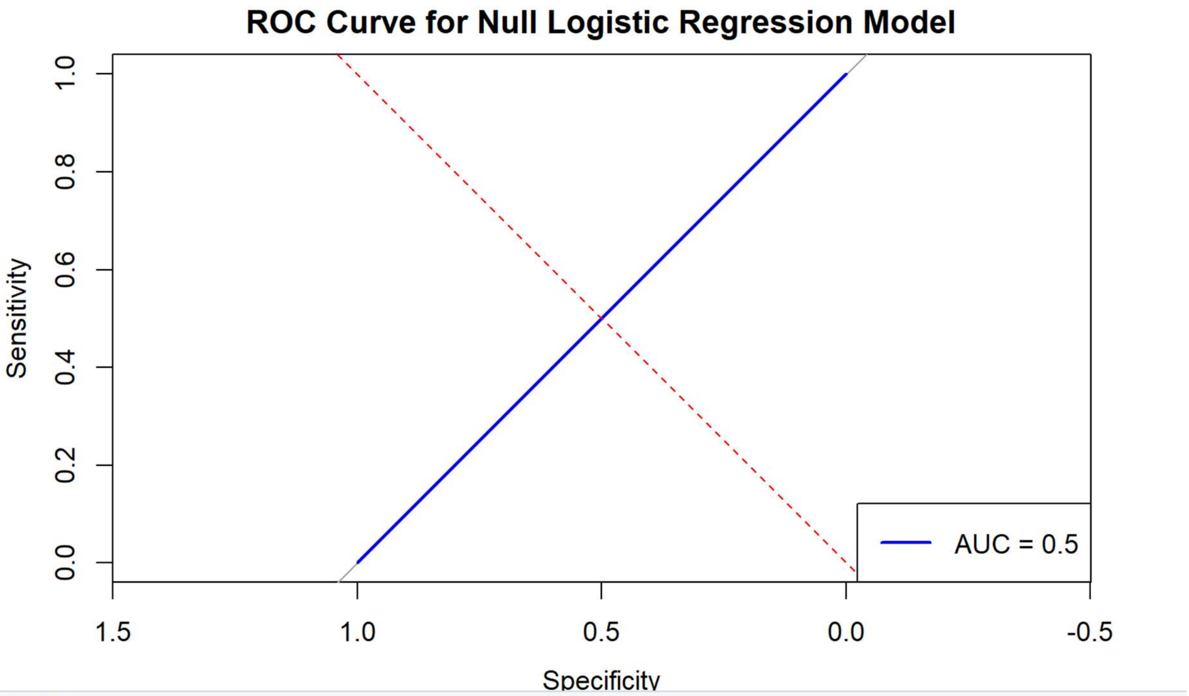

S1 Figure. ROC analysis of Null Model

Full Model and Stepwise Selection

The full model includes a comprehensive set of predictors, including demographic, clinical, and laboratory variables. Forward stepwise selection with AIC was used to identify the best-fit model. This method iteratively adds predictors to minimize the AIC, balancing model fit and complexity.

Final Model (model1) and Interaction Effect

The final model includes significant predictors and an interaction term (`cd4_cat * trans_route`), which was found to be statistically significant ($p = 0.019$). This interaction term indicates that the relationship between the transmission route and the outcome (e.g., mortality) is modified by the CD4 category.

Explanation of Interaction Terms

Interaction terms are used to explore whether the effect of one variable (e.g., transmission route) on the outcome depends on another variable (e.g., CD4 category). A significant interaction term suggests that the predictors do not act independently on the outcome.

For example, in a logistic regression model with an interaction term, the equation is given as:

$$\text{logit}(p) = \beta_0 + \beta_1(\text{cd4\_cat}) + \beta_2(\text{trans\_route}) + \beta_3(\text{cd4\_cat} \times \text{trans\_route})$$

Here, the coefficient β_3 represents the interaction effect, modifying the impact of the predictors on the log-odds of the outcome.

Interpretation of Interaction Effects

For the interaction term `cd4_cat<200 * Other than homosexual`, the odds ratio (OR) is 14.5 (95% CI: 1.76, 169). This indicates that for individuals with CD4 < 200, the odds of the outcome (e.g., mortality) increase 14.5-fold if the transmission route is categorized as 'Other than homosexual' compared to the baseline group (CD4 ≥ 200, Homosexual).

It is important to interpret the interaction effect in conjunction with the main effects to understand the combined influence on the outcome.
